# Insights, beliefs, and myths surrounding tuberculosis among pulmonary patients with delayed healthcare access in a high-burden TB state in Nigeria – a qualitative inquiry

**DOI:** 10.3389/fsoc.2024.1378586

**Published:** 2024-04-17

**Authors:** Beatrice Damilola Adeoye, Turnwait Otu Michael, Richard Dele Agbana

**Affiliations:** ^1^Department of Sociology, Federal University, Oye, Nigeria; ^2^Department of Sociology, University of Johannesburg, Johannesburg, South Africa; ^3^Department of Community Medicine, Afe Babalola University, Ado Ekiti, Nigeria

**Keywords:** tuberculosis, insights, beliefs, myths, pulmonary patients, delayed healthcare access

## Abstract

**Introduction:**

Nigeria grapples with a substantial burden of tuberculosis (TB), particularly in Oyo State, designated as a high-burden State for TB. Effectively addressing this persistent health challenge necessitates more than just medical interventions; it requires a profound understanding of the diverse insights, beliefs, and myths held by TB patients.

**Methods:**

This qualitative study explores the perspectives of pulmonary TB patients with delayed healthcare access in Oyo State, Nigeria, focusing on their beliefs, and conceptions. In-depth interviews were conducted with 25 TB patients and 20 healthcare providers.

**Results:**

Thematic analysis of patients’ responses revealed a complex interplay between cultural, spiritual, and biomedical insights. These challenges questioned the germ theory, associating TB with witchcraft and spiritual attacks. Beliefs in hereditary transmission, links between tobacco use and health outcomes, and uncertainties about infection nature underscored disparities influenced by socio-economic factors. Insights into transmission ideas, preventive measures, and treatment beliefs highlighted a blend of culturally influenced and scientifically supported strategies. Healthcare providers’ insights emphasized the necessity for targeted health education.

**Discussion:**

These findings contribute to a nuanced understanding of TB perceptions, emphasizing the importance of culturally sensitive interventions to enhance awareness and promote timely and accurate health-seeking behaviors.

## 1 Introduction

Tuberculosis (TB) remains a persistent and complicated global health challenge, demanding attention and understanding on multiple fronts (Kidanemariam et al., 2023; Madebo et al., 2023). Caused by the bacterium *Mycobacterium tuberculosis*, this infectious disease primarily targets the lungs but can extend its reach to other organs (Pele et al., 2021; Amare et al., 2022). TB is transmitted through the air, primarily when an infected individual coughs or sneezes, making it a challenging social and public health concern (Datiko et al., 2019; Gopalaswamy et al., 2021). The symptoms of TB range from a persistent cough and chest pain to weight loss, fatigue, and fever (Peirse and Houston, 2017; Field et al., 2018; Long et al., 2020). Despite being treatable with antibiotics, challenges such as drug-resistant strains and societal stigmas surrounding the disease continue to complicate efforts to control its spread (Bashorun et al., 2020; Craciun et al., 2023).

Globally, TB stands as the second most prevalent infectious cause of death, trailing behind COVID-19 (Pan American Health Organization, 2023). In the year 2022, an estimated 10.6 million individuals were afflicted with TB worldwide, encompassing 5.8 million men, 3.5 million women, and 1.3 million children (Pan American Health Organization, 2023; World Health Organization, 2023). TB is pervasive across all nations, with more than 80% of cases and fatalities concentrated in low- and middle-income countries (World Health Organization, 2023). Notably, in 2022, the largest proportion of new TB cases emerged in the South-East Asian Region (46%), followed by the African Region (23%) (Africa Renewal, 2022; World Health Organization, 2023). Nigeria holds the sixth position among the 30 countries with the highest TB burden globally, having the highest burden in Africa and contributing to 4.6% of the global TB load (World Health Organization, 2021).

Tragically, approximately 245,000 Nigerians die from TB annually, with around 590,000 new cases reported. TB alone is responsible for over 10% of all deaths in Nigeria, claiming nearly 30 lives per hour despite the existence of effective treatments (Copenhagen Consensus Center, 2023). The United Nations Sustainable Development Goals (SDGs) include the ambitious health target of eradicating the TB epidemic by 2030, aiming for a 90% reduction in TB-related deaths and an 80% reduction in new cases by the specified year (United Nations, 2015).

Within Nigeria, Oyo State has been identified as a very high-burden region for TB (Akinyemi, 2022; Oguntola, 2023). In the year 2022, the state documented 11,934 confirmed TB cases out of a total presumptive count of 136,222 cases (Oguntola, 2023). Successful TB control initiatives are contingent not only on medical interventions but also on a comprehensive understanding of the diverse factors shaping the lived experiences of those directly affected (Adepoju et al., 2022; Sharma and Khokhar, 2022). Factors contributing to the persistence of TB include socioeconomic disparities, limited access to healthcare, and sociocultural influences that shape community perceptions and behaviors (Du et al., 2022; Olarewaju et al., 2022; Kaaffah et al., 2023).In social psychology, the health belief model posits that individual choices to see health practitioners (registered doctors and nurses) are influenced by perceptions of susceptibility, severity, benefits of action, and barriers to action (Rosenstock, 1974; Limbu et al., 2022). Similarly, the cultural competency theory emphasizes the importance of healthcare professionals acknowledging the ideas and assumptions about health divergent from their own, as well as understanding and respecting cultural beliefs and practices in healthcare delivery (Britni, 2017). In Nigeria, cultural factors such as traditional healing practices and beliefs about the causes of illness influence TB patients’ healthcare-seeking (Asuke et al., 2022; Olarewaju et al., 2022). Policy makers and healthcare providers need to be culturally competent to address these beliefs effectively and provide culturally sensitive care. Oyo State, situated in the southwestern region of Nigeria, mirrors these challenges and serves as a microcosm for understanding the complexities of TB within the broader Nigerian context.

Previous studies have focused on the determinants of TB knowledge, perceptions and treatment adherence, utilizing mainly quantitative approaches (Ojiezeh et al., 2015; Min et al., 2019; Asuke et al., 2022; Chebet et al., 2022; Vericat-Ferrer et al., 2022). The few past studies utilizing qualitative approaches had focused mainly on opinion about TB associated stigma and health seeking behavior (Khan et al., 2020; Huq et al., 2022; Chaychoowong et al., 2023). This study aims to bridge the gap in knowledge by delving into the lived experiences, myths and beliefs among TB patients with delayed access to healthcare in Oyo State. By adopting a qualitative research design, this study seeks to unravel the multifaceted dimensions of insights among patients in Oyo State, exploring the depth of awareness about the causes, symptoms, transmission, and treatment of TB. Attention is also given to examining the sociocultural factors that influence how TB patients interpret and respond to their diagnosis. This is important because ideas about illness can impede effective TB control by fostering fear, stigma, and delayed healthcare-seeking behavior (Ayalew et al., 2020; Onyango et al., 2021). By identifying and understanding these notions, the study aims to contribute insights that can inform targeted awareness campaigns, policies and educational interventions tailored to the specific needs of the study population.

## 2 Materials and methods

### 2.1 Research design

This study adopts a qualitative research design to explore the dimensions of TB-related insights, beliefs, and conceptions among patients in Oyo State. Qualitative methods allowed for an in-depth understanding of patients’ experiences within the sociocultural context. The Standards for Reporting Qualitative Research (SRQR) guided the writing of this manuscript (O’Brien et al., 2014).

### 2.2 Study setting

The research was conducted in various healthcare facilities across Oyo State, encompassing both urban and rural areas, with the aim of capturing diverse perspectives. The selection of these facilities was based on their substantial patient load, ensuring representativeness of the TB patient population. The study area, Oyo State, is situated in the South-West geopolitical zone of Nigeria and was carved out of the former Western Region in 1976. Oyo State is geographically divided into five zones: Ibadan Areas (11 Local Government Areas - LGAs), Oke-Ogun Areas (10 LGAs), Ogbomoso Areas (5 LGAs), Oyo Areas (4 LGAs), and Ibarapa Areas (3 LGAs), totaling 33 local government areas. With a population of 7,512,855 people and covering a landmass of 28,245.26 square kilometers (National Bureau of Statistics, 2020), Oyo State is marked by rich traditions and cultural beliefs. The profound influence of religious and traditional beliefs within the population is evident through the prevalence of various religious and traditional healing establishments offering healthcare services. The majority of residents in the State adhere to either Christianity or Islam. It is noteworthy that Oyo State is among the three states in Nigeria with the highest TB prevalence rate, underscoring the significance of understanding TB-related knowledge, perceptions, beliefs, and conceptions in this particular region. The diverse geographic and cultural landscape of Oyo State provides a unique context for exploring the complexities of TB experiences among its residents (Ojiezeh et al., 2015).

### 2.3 Sampling

Purposive sampling was utilized to select pulmonary TB patients undergoing treatment in the designated health facilities with evidence of delayed healthcare access. The participants were deliberately chosen to ensure a diverse representation across various factors, including age, gender, socioeconomic status, and geographic location within Oyo State. In-depth interviews were conducted with two distinct categories of respondents: healthcare providers and TB patients, using a purposive sampling method. The first category comprised 20 healthcare providers, predominantly nurses and doctors (15 nurses and 5 doctors), directly involved in the treatment of TB patients. These practitioners provided accurate and valuable information essential for this research. Specifically, 5 healthcare providers were selected from directly observed treatment (DOT) centers, 5 from state hospitals, and 10 from the University College Hospital (UCH), a prominent federal teaching hospital.

The second category of interview respondents consisted of pulmonary TB patients with records of delayed healthcare access to facility for TB treatment. In general, guidelines recommend that individuals seek medical evaluation promptly if they experience symptoms suggestive of TB, such as persistent cough, fever, night sweats, weight loss, and fatigue. Delays in seeking care beyond a few weeks to a month after the onset of symptoms is an indicative of delayed healthcare seeking (Centers for Disease Control and Prevention, 2005, 2023b). However, this timeframe may vary depending on the severity and nature of the symptoms, hence we relied on health facility records (Centers for Disease Control and Prevention, 2005), A total of 25 TB patients were interviewed, drawn from the University College Hospital (UCH-Ibadan) and Oyo State government hospitals (Adeoyo and Oluyoro Hospital). Among these, 15 patients were selected from UCH federal teaching hospital, and 10 patients, comprising 5 from Oyo State government hospitals, Adeoyo and Oluyoro Hospital, respectively. The decision to focus on federal and state hospitals was driven by the belief that these institutions would yield a sufficient number of patients capable of providing comprehensive information. In total, 45 respondents (20 healthcare providers directly involved in TB treatment and 25 TB patients) participated in the in-depth interviews using structured guides. While patients provided information about their perspectives on TB, the healthcare providers shared insights about accurate information and appropriate medical interventions, diagnoses, treatments, and symptoms of TB. Additionally, they conveyed general information, including ethical considerations, about TB patients’ reasons for delaying visits to healthcare facilities for diagnosis and treatment. Their responses aided in assessing the alignment of patients’ beliefs, myths, and information regarding TB with medical perspectives. The determination of the sample size was guided by the principle of data saturation, ensuring that the inclusion of additional interviews did not yield new themes or insights.

### 2.4 Data collection

In-depth Interviews were conducted with both pulmonary TB patients and healthcare providers to explore their knowledge, perceptions, beliefs, and experiences related to TB. Semi-structured interview guides were employed to cover essential topics while providing flexibility for participants to express their unique perspectives. Participants granted consent for the interviews, which were audio-recorded. The interviews were facilitated by the researchers with the support of a trained research assistant at each study location to ensure the collection of accurate and comprehensive information. For the in-depth interviews with TB patients, precautions were taken to prevent the potential transmission of TB infections. Health professionals guided researchers to position themselves not directly in front of TB patients but by their side, preferably in well-ventilated areas. Additionally, nose masks were provided to the researchers in certain instances. Interview questions included “What do you think are the causes of TB? What are TB modes of transmission? How can TB be prevented? Where do you think TB can be best treated and why?” Conducting interviews with TB patients posed challenges as a significant number were apprehensive about potential stigmatization and expressed anger about their situation. Some initially hesitated to participate in the interviews. To address these concerns, researchers actively encouraged and assured participants of the confidentiality of their responses, fostering a cooperative and trusting environment for meaningful engagement in the research process.

### 2.5 Data analysis

The audio-recorded interviews were transcribed verbatim, and anonymized for analysis Thematic analysis was employed to identify patterns, codes, and themes within the qualitative data (Castleberry and Nolen, 2018). Transcripts were independently coded by two researchers, and regular meetings were held to discuss emerging themes and resolve discrepancies. Themes were organized to capture the complexity of TB-related knowledge, perceptions, beliefs, and notions. The study utilized ATLAS.ti, a computer-aided software for qualitative data analysis. The process included a multi-step approach to gain insights into TB beliefs and myths. Initially, researchers composed memos and reflections. Subsequent readings of transcripts unveiled significant patterns, coded and organized into themes and subthemes. This iterative process allowed for the extraction of meaningful insights and exploration of connections between variables of interest. Beginning with the generation of initial codes, essential concepts within the data were succinctly summarized. Open coding segmented data into meaningful units, assigning codes to each segment. Relationships between codes were scrutinized, leading to the grouping of similar codes into categories and the derivation of primary themes through the exploration of their connections. Utilizing these categories, themes emerged, reflecting central ideas. Sub-themes within these larger themes provided in-depth insights. Themes underwent review, complemented by detailed descriptions and narratives to enhance understanding. A comprehensive thematic analysis report was compiled, encompassing primary themes, sub-themes, descriptive narratives, and interpretive insights. Direct participant quotes were included to add authenticity. Additionally, peer review feedback from colleagues and subject matter experts reinforced the analysis’s rigor. Following expert feedback, the themes were revised and finalized, as presented in the manuscript’s results section, shedding light on the complex landscape of TB beliefs and myths among pulmonary patients in Nigeria (Michael, 2024).

### 2.6 Ethical considerations

This research adhered to the ethical principles outlined in the Declaration of Helsinki for research involving human subjects. Prior to data collection, the study was thoroughly explained to recruited respondents, and written consent was obtained from each participant. Informed consent underscored the voluntary nature of participation and ensured the confidentiality of their information. Ethical approval was secured from the Ethics and Research Review Board of Federal University, Oye-Ekiti, Nigeria (Reference Number: FUOYE/SOC/ETHICS/002). To protect participants’ identities, pseudonyms and unique codes were employed during data analysis and reporting.

### 2.7 Reflexivity

Researchers maintained reflexivity throughout the study, acknowledging their own biases and preconceptions (Wilson et al., 2022). Reflexivity was documented to enhance transparency and rigor. In the context of the study, researchers openly acknowledged and reflected upon their biases, assumptions, and beliefs regarding TB. This involved recognizing any preconceived notions about TB patients, their communities, or the effectiveness of existing TB control programs that might impact the research process. Reflexivity was crucial in this study because the first and second authors, both sociologists, collaborated with the third author, a medical doctor whose expertise lies in the biomedical domain, hence having insights rooted in the medical scientific understanding of TB, including methods of prevention and treatment. Reflexivity further informed the interactions between researchers and participants during data collection, ensuring that the researchers’ presence did not unduly influence participants’ responses. Researchers strived to maintain an open and non-judgmental attitude during interviews. Reflexivity extends to the data analysis phase, where researchers critically examined their role in shaping the interpretation of findings. The researchers actively engaged in discussions to challenge and validate their interpretations, reducing the risk of biased conclusions. Researchers maintained a reflexive record, documenting insights, challenges, and personal reflections throughout the research process. This documentation served as a reference point for understanding how the researchers’ perspectives may have influenced decisions and interpretations. By openly addressing their own subjectivities and presenting findings without biases, researchers contributed to the overall trustworthiness and credibility of the study (Yip, 2023).

## 3 Results

### 3.1 Socio-demographic characteristics of the participants

The socio-demographic characteristics of the participants are illustrated in Table 1. In relation to gender, the patient group comprises 14 males, while the healthcare provider group consists of 13 females. Educational backgrounds display variations, with 12 patients having a secondary education. Concerning residence, 15 patients reside in rural areas. Healthcare providers are evenly distributed between rural and urban residences, with 10 in each category. Regarding professional categories, nurses constitute 15 of the healthcare providers.

**Table 1:** Socio-demographic characteristics of the participants.

Variables	Patients (P)	Healthcare providers (HCP)
Sex		
Male	14	7
Female	11	13
Education		
Primary	6	–
Secondary	12	–
Tertiary	7	20
Residence		
Rural	15	10
Urban	10	10
Category		
Doctors	–	5
Nurses	–	15
Total	25	20

P, Patient; HCP, Healthcare provider.

### 3.2 Theme 1: insights and beliefs about the causal factors for TB

**Figure 1:**
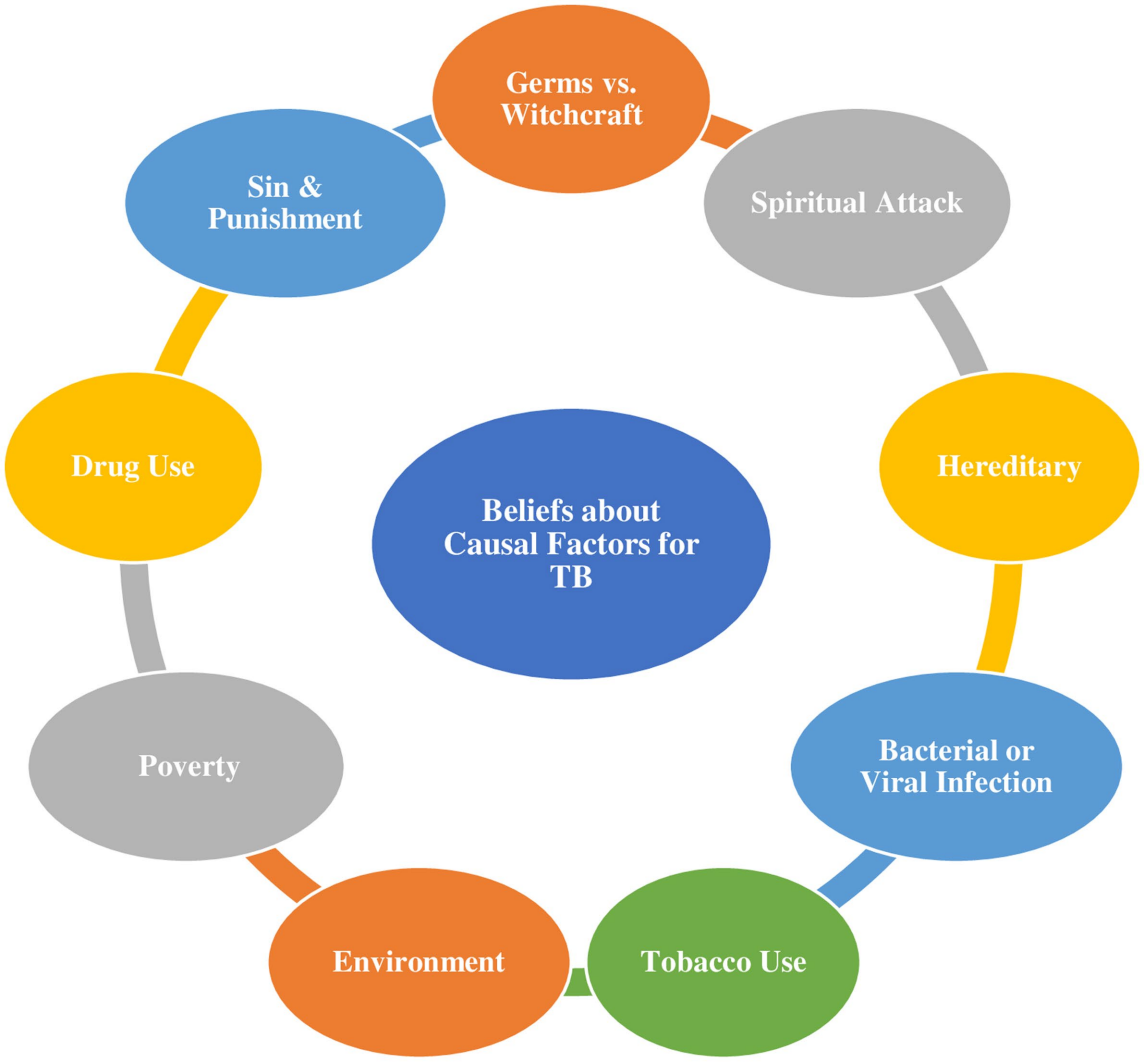
Schematic illustration of participants’ beliefs about the casual factors for TB.

The interview transcripts reveal a diverse array of beliefs regarding the cause of TB among the respondents, encompassing spiritual attacks, hereditary influences, punishment for wrongdoing, bacterial or viral infections, tobacco use, and environmental factors (Figure 1).

#### 3.2.1 Local names, germs versus witchcraft

The interviews with TB patients also highlight the diverse local names attributed to TB within the community. More than half of the respondents (52%) in the study commonly identify TB as Ikofe, translating to “Dry Cough.” Additionally, 28% refer to TB as Iko Jedojedo, meaning “Cough that eats up the liver,” 12% recognize it as Iko Awule, describing “Cough that causes people to emaciate,” and 8% associate TB with Iko Eleje, signifying “Bloody Cough.” This indicates that participants characterize TB based on their understanding of the effects or damages it may cause in the body.

Notably, some participants challenge the widely accepted germ theory by attributing TB to witchcraft, positing that germs are not the cause, but rather the disease emanates from spiritual sources. This is exemplified by the assertions, “*Germs do not cause TB… Witchcraft is the source of TB” (P5).* The participants link the onset of their TB to spiritual attacks, correlating coughing episodes with recent deliverance ceremonies and suggesting the possibility of TB being cast as a spell by malevolent individuals.

*“In my TB case, I knew it was a spirit world attack since the cough did not start until 3 days after we performed a deliverance for someone.” (P2). “I know TB has something to do with coughing, and it may be cast as a spell on someone by those who are evil.” (P3)*.

#### 3.2.2 Hereditary, drug/tobacco use, and infections

Furthermore, the belief in hereditary transmission of TB is emphasized, with participants drawing connections between their own cases and family histories of the disease. Statements such as “*TB is inherited, meaning that it runs in families… I am not surprised by the cause of my TB because it runs in my family” (P14)* underscore the perception that if one family member has TB, others within the family may also be susceptible. According to participants, this belief aligns with the practice of doctors inquiring about family history during medical consultations, emphasizing the significance placed on familial connections in understanding TB susceptibility.

*“Because TB is a hereditary disease, if one person in a family gets it, another person in the family may also have it; which is why hospitals usually ask about your family history and whether anyone in your family has ever had TB.” (P21).*

Tobacco use is intricately linked to the participants’ conceptualization of TB, with an emphasis on smoking history as a diagnostic factor for TB. Additionally, a distinctive perspective emerges, associating TB with the hereditary transmission of punishment for violating traditional rules, indicating a complex intertwining of cultural and health-related beliefs. *“TB is hereditary, meaning it is passed down from one person in a family line who has committed a crime. The main cause of TB can be traced back to punishment for violating traditional rules and regulations.” (P8).* The statements, “*Tobacco use causes TB, which is where the name tuberculosis comes from” (P17)*, highlight the intricate connections drawn between cultural practices, substance use, and health outcomes.

While some participants accurately attribute TB to infection, the specific nature of the infection (bacterial or viral) remains uncertain in their understanding. *“An infection causes TB. I just do not know if it’s caused by a bacterial or viral infection. But I know it is caused by an infection that is breathed through the air.” (P9).* Additionally, the interviews reveal a prevailing perception of TB as a disease of the impoverished, linked to an unclean environment and poverty. This belief suggests that maintaining a healthy lifestyle through cleanliness and proper nutrition is seen as a preventive measure against TB. *“TB is a disease of the poor since it is caused by an unclean environment and poverty. You will not get TB if you eat healthy and live in a clean environment.” (P20).*

Personal experiences with substance use contribute to the participants’ association of TB primarily with drug use. “…*I know my tuberculosis is caused by the harsh drugs I use.” (P11).*

#### 3.2.3 Sin, punishment, and spiritual attack

The interviews further unveil a belief in sin as the root cause of TB, aligning it with a form of divine punishment analogous to HIV/AIDS. This spiritual perspective is captured in the statements, *“Sin is the root cause of TB. It is punishment for wrongdoing. God sends diseases like tuberculosis and HIV/AIDS to punish sinners.” (P22)*, underlining the influence of cultural and religious beliefs in shaping perceptions of health and illness.

Interestingly, despite the prevalence of spiritual explanations, some participants acknowledge bacterial or germ infections as the cause of TB, demonstrating a divergence in beliefs within the community. *TB is caused by bacteria or germs that have infected us and have remained in our bodies for an extended period of time without being treated.” (P16).* Healthcare professionals, particularly a doctor and a nurse, shed light on the community’s inclination to perceive TB more as a spiritual than a physical ailment.

This perception, as mentioned by a doctor, contributes to delayed hospital visits among residents who frequently attribute the disease to spiritual causes. A nurse notes that patients often believe TB is an intentional attack from adversaries. Another nurse highlights the common assumption by patients that TB has genetic roots, prompting individuals to trace it back to their parents or family members.

*“People in this area believed that TB was more spiritual than physical, which is why they frequently delayed seeking treatment at the hospital.” (HCP, Doctor 3). “…they believe that tuberculosis is an attack from their enemies.” (HCP, Nurse 1). People assume that tuberculosis is genetic and frequently want to trace it back to their parents or family members.” (HCP, Nurse 9)*.

### 3.3 Theme 2: transmission and myths surrounding TB

**Figure 2:**
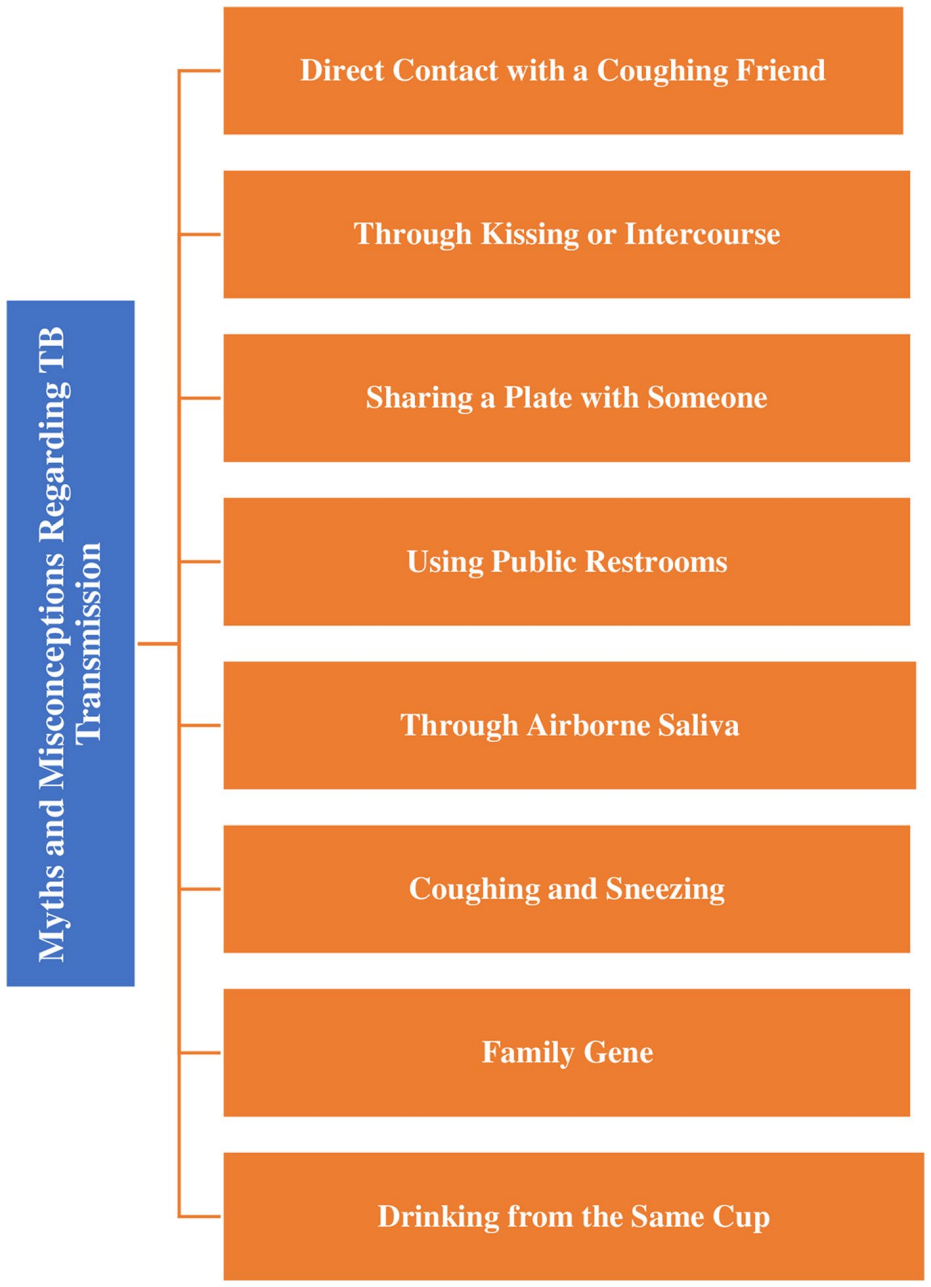
Schematic illustration of myths and misconceptions regarding transmission of TB.

The interview transcriptions provide insights into a spectrum of beliefs and conceptions surrounding the transmission of TB, reflecting the impact of cultural perspectives and individual experiences, as shown in Figure 2.

#### 3.3.1 Contact with a coughing friend, kissing and intercourse

A participant attributes their TB infection to close interaction with a coughing friend, aligning with the recognized mode of transmission through respiratory droplets. The statement, “*My tuberculosis was caught through interaction with a coughing friend. I later discovered he had tuberculosis” (P14),* illustrates a common scenario where individuals unknowingly expose themselves to the bacteria during close contact.

Also, the introduction of the belief that “*kissing or having intercourse with someone with tuberculosis can lead to TB infection (P5)* oversimplifies the primary mode of transmission, which is through inhaling respiratory droplets. This notion may contribute to stigma surrounding the disease, highlighting the need for accurate information dissemination and education.

#### 3.3.2 Sharing plate, public restroom, and airborne saliva

The interviewees express a belief that sharing a plate with someone who has TB may result in contracting the disease. The statement*, “If you share a plate with someone who has tuberculosis, you may contract the disease. That is why individuals isolate themselves from people who have tuberculosis. I no longer advise my children to come near me” (P16),* reveals a misunderstanding of the primary mode of TB spread. The decision to isolate oneself and advise children to stay away underscores the fear and stigma associated with TB, emphasizing the importance of addressing misconceptions to reduce social isolation.

Another notion introduced by participants is the belief that sharing a public restroom or toilet can expose individuals to TB. This idea is medically unfounded, as the primary mode of transmission is through airborne respiratory droplets, not casual contact in shared spaces. The statement, “*Sharing a public restroom or toilet with others can expose you to tuberculosis” (P1),* emphasizes the need for public health education to dispel misconceptions and foster an accurate understanding of TB transmission dynamics.

Participants also introduce the belief that airborne saliva in a room where someone has TB can lead to infection. While TB is an airborne disease, the likelihood of transmission through airborne saliva is minimal. The statement, “*By spitting forth saliva. If you enter into a room where someone has TB and you do not know who it is, and the saliva is still in the air, you can contact TB” (P3),* may contribute to heightened anxiety and precautions, emphasizing the importance of clear communication to address fears grounded in the belief about TB.

#### 3.3.3 Family gene and drinking from the same cup

Additionally, participants express the belief that TB can be contracted through a family gene, suggesting hereditary transmission. While there is a genetic component influencing susceptibility, TB is primarily an infectious disease. This belief may contribute to fears about familial transmission and underscores the importance of addressing genetic conceptions surrounding TB. The statement: “*TB can be contacted through a family gene. It’s in the blood. Because I inherited it from my parents, one or two of my children may develop tuberculosis in the future.” (P22).*

The introduction of the belief that sharing a cup with a TB-infected person can lead to infection is not supported by medical evidence, as TB is primarily transmitted through respiratory droplets. The statement, “*If a TB-infected person drinks water from a cup and you drink water from the same cup, you can catch TB. As a result, it is not advisable to share a cup with someone you do not trust. I’d like to think that’s how I got TB” (P3),* reflects a perception of potential contagion, highlighting the need for clear communication to dispel unfounded fears.

Amidst these ideas, some participants’ statements align with accurate information, recognizing that TB is spread by infected individuals coughing or sneezing. The cultural practice of keeping one’s mouth closed during coughing or sneezing reflects an understanding of preventive measures to reduce the risk of transmission. *TB is spread by another infected individual coughing or sneezing. As a result, in our culture, everyone is instructed to keep their mouth closed when coughing or sneezing.” (P19).* This statement exemplifies the potential for cultural practices to align with evidence-based public health recommendations, emphasizing the importance of incorporating cultural sensitivity in health education initiatives.

### 3.4 Theme 3: TB prevention strategies: insights from interviews

**Figure 3:**
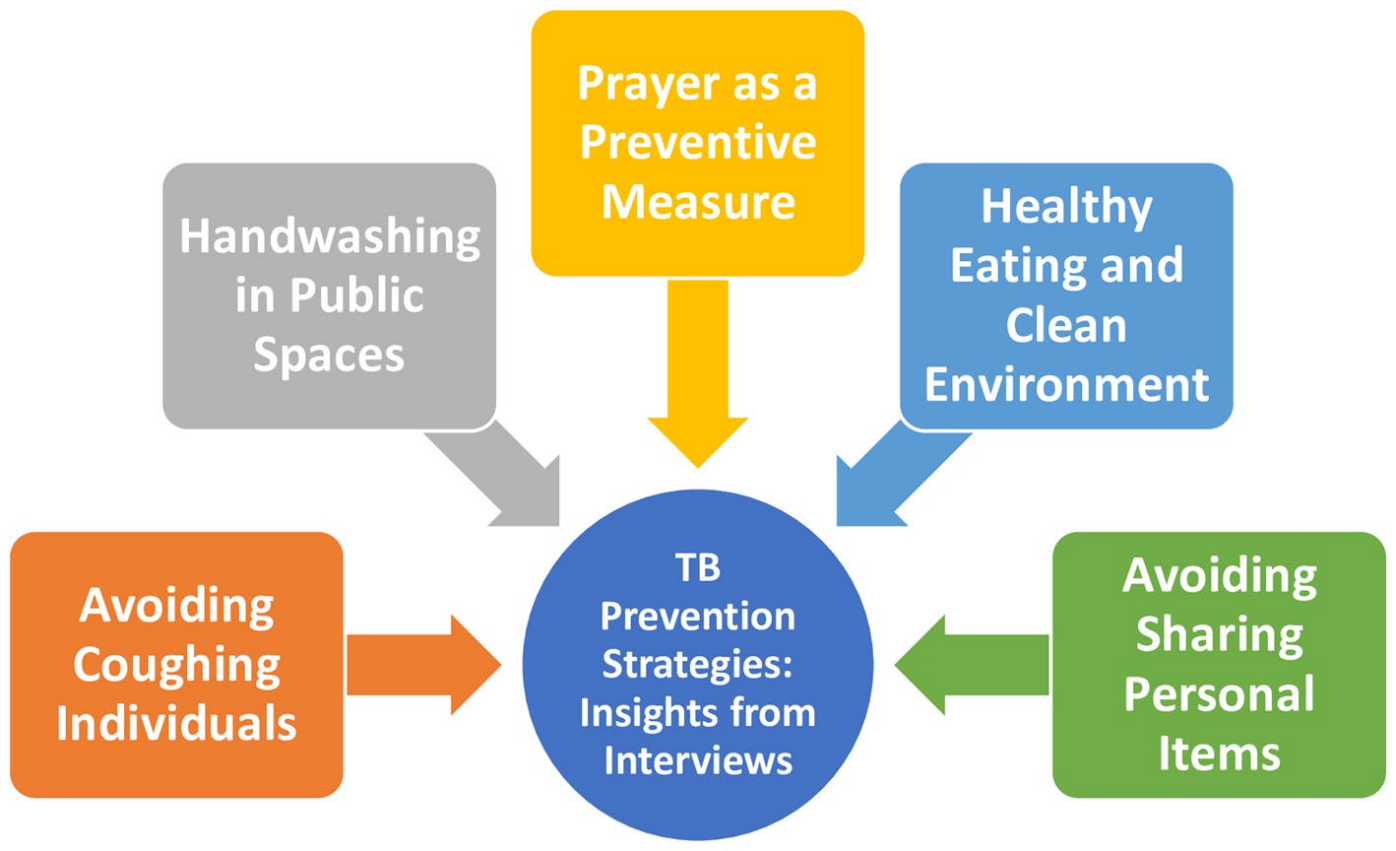
Schematic of participants’ beliefs about TB prevention.

The interviews conducted reveal a range of beliefs and practices related to TB (TB) prevention among participants (see Figure 3).

#### 3.4.1 Avoiding coughing individuals and handwashing in public spaces

Patients with TB suggest avoiding proximity to someone who is coughing as a preventive measure: “*To avoid TB, avoid being around somebody who is coughing because you do not know how long the individual has been coughing or if the person has TB” (P2).* This advice aligns with scientifically supported preventive measures, given that TB is primarily transmitted through respiratory droplets. However, it’s crucial to note that individuals with latent TB infection may not display symptoms, and solely avoiding those who cough may not entirely eliminate the risk. Nevertheless, the recommendation demonstrates an awareness of the respiratory route of transmission.

Participants emphasize the importance of hand hygiene, suggesting that hands should be washed after touching items in public due to concerns about potential contact with surfaces touched by a TB patient: *“Always wash your hands after touching items in public since it could have been touched by a TB patient” (P23).* While TB is not typically transmitted through surface contact, promoting good hand hygiene is a positive measure to prevent the spread of various infections, including respiratory ones.

#### 3.4.2 Prayer, healthy eating and clean environment

Spiritual beliefs are introduced as a preventive measure, with participants expressing faith in the power of prayer to avoid TB: *“There is nothing that prayer cannot achieve. Always pray to avoid tuberculosis. Pray before leaving your house, while outside, and when you return home to keep diseases away” (P4)*. While prayer can provide emotional support, it is not scientifically proven to prevent TB. The recommendation underscores the importance of combining spiritual beliefs with evidence-based practices, such as vaccination, hygiene, and avoiding close contact with infected individuals.

The connection between healthy eating, a clean environment, and TB prevention is highlighted, with participants attributing the disease to poverty: *“To prevent tuberculosis, eat healthily and live in a clean and sanitary environment because TB is a poor people’s illness, which is why it is common in poor nations” (P25).* While malnutrition and poor living conditions can weaken the immune system, making individuals more susceptible to infections, TB is not exclusive to poor nations. The advice to eat healthily and maintain a clean environment is generally sound for overall well-being but should be supplemented with other evidence-based preventive measures.

#### 3.4.3 Avoiding sharing personal items

Participants also stress the importance of not sharing personal items, such as drinking cups and eating plates, to avoid TB:

*“To avoid tuberculosis, avoid sharing personal items with strangers. Drinking cups and eating plates should not be shared. Eat from different plates” (P1)*.

This recommendation aligns with good hygiene practices and may help reduce the risk of various infectious diseases, including TB. While TB is not typically transmitted through shared utensils, the emphasis on avoiding sharing personal items reflects a cautious approach to minimize potential exposure.

### 3.5 Theme 4: TB treatment perspectives: insights from interviews

**Figure 4:**
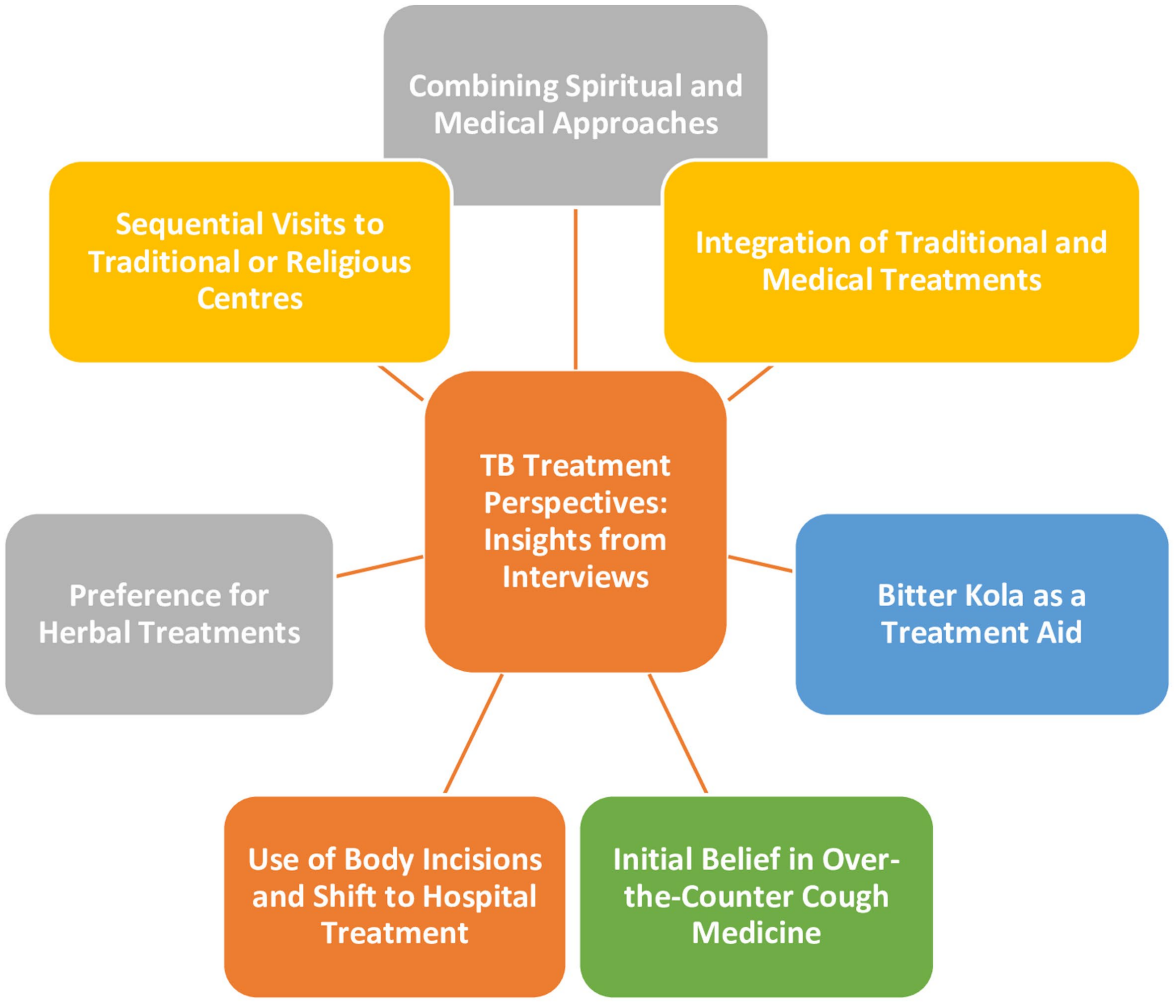
Schematic of participants’ perspectives about TB treatment.

Interviews with TB patients shed light on diverse beliefs and treatment approaches related to TB, reflecting a complex interplay of traditional, spiritual, and medical practices (Figure 4).

#### 3.5.1 Combining spiritual and medical approaches

The participants stated that there is no single approach to treating TB because the root cause of the condition may be either spiritual or physical, or perhaps a combination of both. The interviewees mention a multifaceted approach to seeking treatment. In addition to going to the hospital for medical care, they also incorporate spiritual practices by visiting a prayer house. Additionally, they mentioned using holy water and olive oil, indicating the inclusion of religious or spiritual rituals as part of their approach to managing TB. This reflects a belief in the interconnectedness of physical and spiritual aspects in dealing with the illness: *“There is no one approach to treat tuberculosis because you may not know the specific root of the condition, whether spiritual or physical. When I go to the hospital, I also go to the prayer house. I also make use of holy water and olive oil” (P2).* This amalgamation of traditional, spiritual, and medical elements in their treatment plan illustrates the multifaceted nature of their approach.

Advocates for combining traditional and medical treatments are prevalent among participants, emphasizing the incorporation of local herbal remedies such as ginger, garlic, and alligator pepper: *“To cure tuberculosis, both traditional and medical treatments are required. You must drink local medications manufactured from herbs while receiving hospital care. Ginger, garlic, and alligator pepper are also useful in the treatment of tuberculosis. I do the same” (P8).* While the cultural significance of traditional practices is evident, the effectiveness of such treatments warrants scientific validation to ensure safe and evidence-based care.

#### 3.5.2 Bitter kola and body incisions as treatment aids

Bitter kola is perceived as aiding in TB treatment, with participants consuming it alongside prescribed medications*: “Bitter cola aids in the treatment of tuberculosis. Eat extra of it while taking the medications prescribed by your doctor, that is what I do” (P24).* This belief in the medicinal properties of bitter kola is rooted in traditional practices, highlighting the need for scientific scrutiny to evaluate its overall efficacy.

The use of body incisions for treating TB, particularly when perceived as genetic or a result of a spiritual attack, is mentioned. The recognition that spiritual attacks require different treatment approaches, coupled with the pragmatic shift to hospital care, demonstrates an evolving understanding of evidence-based interventions: *“We use body incisions to treat tuberculosis, especially when it is genetic or the result of a spiritual attack. Spiritual attacks are not treated with hospital medicine. I’m in the hospital because I discovered that the incision does not work, indicating that my TB is not a spiritual attack” (P6).*

While some participants believe herbs and concoctions treat TB faster than hospital drugs, they acknowledge the challenge of finding trustworthy herbalists. Seeking hospital treatment reflects a pragmatic response to concerns about fraudulent practices: “*Herbs and concoctions treat tuberculosis faster than hospital drugs. Finding a good herbalist is now difficult. We now have a lot of fake herbalists, which is why I’m in the hospital for treatment” (P17).*

#### 3.5.3 Belief in over-the-counter cough medicine and religious centers

The interviews also uncover a common notion among TB patients who initially believed that any antibiotic cough medicine could cure TB. The subsequent realization of the necessity of hospital drugs highlights the critical role of evidence-based medical interventions:

*“I used to believe that any antibiotic cough medicine might heal tuberculosis. I was taking cough medicine that I had purchased from a pharmacy. When that did not work, I went to the hospital. To cure tuberculosis, you must use hospital drugs” (P5).*

Healthcare providers acknowledge that many TB patients initially visit traditional or religious centers, potentially delaying hospital treatment. This delay underscores the need for improved health education and community outreach to promote timely medical intervention: *“When other forms of treatment failed to cure the ailments, the majority of TB patients who visited the hospital first visited traditional or religious centers before coming to the hospital for treatment” (HCP, Doctor 5).*

Additionally, healthcare providers note that most TB patients arrive at the hospital late due to beliefs in self-medication. This observation emphasizes the urgent need to address conceptions and promote early medical intervention through effective public health campaigns: *“Most TB patients arrive at the hospital late because they believe they can cure the disease with remedies or self-medication” (HCP, Nurse 14).*

## 4 Discussion

Participants challenging the germ theory and attributing TB to witchcraft highlight the imperative for culturally sensitive health communication, aiming to reconcile traditional beliefs with biomedical knowledge (Brooks et al., 2019). Spiritual explanations for TB, intertwined with cultural practices like correlating coughing episodes with deliverance ceremonies, echo findings from other regions, like Eritrea, where a significant percentage of respondents (79.8%) did not associate TB with bacteria/germs (Kidanemariam et al., 2023). The influence of education on TB knowledge aligns with studies in China and Indonesia (Du et al., 2022; Kaaffah et al., 2023).

The belief in hereditary transmission resonates with familial connections, emphasizing the importance of addressing concerns and promoting accurate understanding of genetic components in TB. Associations between tobacco use, cultural practices, and health outcomes mirror previous findings linking tobacco smoking to TB (Wang et al., 2018). The uncertainty about the specific nature of infection (bacterial or viral) highlights a crucial knowledge gap that necessitates targeted education. The perception of TB as a disease linked to poverty emphasizes socio-economic influences on health beliefs, contrasting findings from South Africa (Onyango et al., 2021). Associations between substance use and sin as TB causes underscore the complex interplay of cultural and religious beliefs, deviating from the results of a study conducted in Limpopo Province (Matakanye et al., 2021). In support of the social determinant of health theory, this perspective highlights how social factors such as poverty, education, beliefs, and access to healthcare services influence health outcomes (Dean et al., 2013). In Nigeria, socio-economic disparities contribute to delayed healthcare access for TB patients (Onyango et al., 2021). For instance, impoverished individuals may lack resources to seek timely medical care, while limited healthcare infrastructure in rural areas exacerbates accessibility issues.

Participants acknowledging bacterial or germ infections demonstrate a divergence in beliefs within the community, emphasizing the need for nuanced health communication and intervention. Insights from healthcare professionals about the community’s inclination to perceive TB as more spiritual than physical underscore potential barriers to timely medical intervention. Addressing these notions through targeted health education, cultural and behavioral improvements are critical (Adepoju et al., 2022; Amare et al., 2022). This is essential because according to health belief model, individual health behaviors are influenced by perceptions of susceptibility, severity, benefits of action, and barriers to action (Limbu et al., 2022). Similarly, in the study, cultural beliefs and myths surrounding TB influence individuals’ perceptions of susceptibility and severity, leading to delays in seeking healthcare. Stigma associated with TB and conceptions about its transmission also act as barriers to seeking timely diagnosis and treatment.

Conceptions and ideas about TB transmission through kissing, sharing plates, using public restrooms, and exposure to airborne saliva highlight the importance of accurate information dissemination to reduce stigma. Our findings align with studies in Indonesia, emphasizing cultural practices’ role in TB transmission (Pele et al., 2021). Participants’ beliefs about genetic transmission and the need for isolation reflect underlying fears and misconceptions. Harmonizing cultural practices with evidence-based public health recommendations is vital (Centers for Disease Control and Prevention, 2023a). TB prevention beliefs and practices showcase a blend of culturally influenced measures and scientifically supported strategies. Advocacy for avoiding proximity to coughing individuals aligns with recognized preventive measures. While emphasizing hand hygiene may not directly relate to TB, it promotes general infection control practices (Centers for Disease Control and Prevention, 2022).

The inclusion of prayer as a preventive measure highlights the intersection of spiritual and health beliefs. Combining spiritual beliefs with evidence-based practices offers a holistic approach to address physical and emotional well-being. The connection between healthy eating, a clean environment, and TB prevention underscores the importance of comprehensive well-being (Centers for Disease Control and Prevention, 2023a). Emphasizing not sharing personal items aligns with good hygiene practices but dispelling conceptions about TB transmission through shared utensils is crucial (Madebo et al., 2023). Our interviews with TB patients uncover a diverse range of treatment beliefs and approaches, emphasizing the complex interplay of traditional, spiritual, and medical practices. The amalgamation of these elements underscores the need for culturally sensitive healthcare. Advocacy for combining traditional and medical treatments, incorporating local herbal remedies, and the perceived benefits of bitter kola warrant scientific scrutiny for safe and evidence-based care. Acknowledgment of the ineffectiveness of body incisions for spiritual attacks indicates a pragmatic shift toward evidence-based treatment (Audet et al., 2023).

Challenges in finding trustworthy herbalists and the recognition of the necessity for hospital drugs underscore a critical need for accurate health information dissemination. The conception about over-the-counter cough medicine and the subsequent realization of the necessity for hospital drugs highlight the importance of promoting accurate health-seeking behaviors (Khan et al., 2020). The delay in hospital visits due to initial visits to traditional or religious centers emphasizes the need for improved health education and community outreach. Addressing misconceptions and promoting early medical intervention is crucial for reducing disease severity and transmission risk (Onyango et al., 2021). Our findings align with studies in Nigeria, emphasizing patient-related diagnostic delays associated with education, religion, and healthcare workers’ attitudes (Olarewaju et al., 2022; Michael et al., 2023).

## 5 Research and policy implications

Given the diverse array of beliefs and perceptions uncovered, future research should focus on developing and implementing culturally sensitive health interventions. Tailoring education and awareness programs to local beliefs and practices could bridge the gap between traditional perspectives and biomedical knowledge, facilitating more effective TB prevention and control strategies. The identified knowledge gaps, such as uncertainties about the nature of TB infection, underscore the need for targeted educational campaigns. Research should explore innovative ways to enhance community understanding of TB, particularly focusing on the realities about the disease and dispelling misconceptions.

Policymakers should consider incorporating cultural competence into TB control policies. Recognizing and respecting local beliefs can enhance the acceptability and effectiveness of health interventions. Collaborations between health authorities and community leaders can facilitate the development of culturally sensitive policies. Policies should prioritize the integration of robust health education programs that target both urban and rural communities. These programs should emphasize accurate information dissemination about TB transmission, prevention, and treatment, aligning with the diverse cultural and educational backgrounds of the population. Implementing community-based TB screening programs can enhance early detection and intervention. Policy efforts should focus on decentralizing testing services and ensuring accessibility, especially in remote areas, to reduce diagnostic delays and improve patient outcomes. Given the prevalent use of traditional and herbal remedies, policies should explore the integration of traditional healers into the broader healthcare system. Collaborative efforts between traditional healers and medical professionals could lead to a more comprehensive and culturally aligned approach to TB care.

## 6 Conclusion

Our study provides valuable insights into the complex dynamics of TB perceptions, transmission beliefs, and treatment approaches within the community. Bridging the gap between cultural beliefs and biomedical knowledge, dispelling misconceptions, and promoting evidence-based practices are crucial for effective sociological and public health interventions. Culturally sensitive health education initiatives are necessary to foster accurate understanding, reduce stigma, and encourage timely medical intervention for TB prevention and treatment. This includes training healthcare providers in cultural competency to understand and respect local beliefs, engaging community leaders and traditional healers to dispel myths, creating educational materials in local languages, organizing interactive workshops and discussions, establishing peer support networks, and integrating TB services into existing healthcare facilities. For instance, healthcare providers could undergo training to understand and address cultural beliefs about TB, such as involving traditional healers in TB education campaigns or creating community-led support groups for TB patients. These efforts would help to bridge the gap between medical knowledge and community beliefs, ultimately improving TB prevention, diagnosis, and treatment outcomes in culturally sensitive ways.

## Data availability statement

The original contributions presented in the study are included in the article/supplementary material, further inquiries can be directed to the corresponding author.

## Ethics statement

The studies involving humans were approved by Ethics and Research Review Board of Federal University, Oye-Ekiti, Nigeria. The studies were conducted in accordance with the local legislation and institutional requirements. The participants provided their written informed consent to participate in this study.

## Author contributions

BA: Conceptualization, Data curation, Investigation, Methodology, Project administration, Resources, Software, Supervision, Validation, Writing – original draft, Writing – review & editing. TM: Conceptualization, Data curation, Formal analysis, Methodology, Resources, Software, Supervision, Validation, Visualization, Writing – original draft, Writing – review & editing. RA: Investigation, Methodology, Resources, Supervision, Validation, Visualization, Writing – original draft, Writing – review & editing.
